# Exogenous γ-Aminobutyric Acid Improves the Structure and Function of Photosystem II in Muskmelon Seedlings Exposed to Salinity-Alkalinity Stress

**DOI:** 10.1371/journal.pone.0164847

**Published:** 2016-10-20

**Authors:** Lixia Xiang, Lipan Hu, Weinan Xu, Ai Zhen, Liang Zhang, Xiaohui Hu

**Affiliations:** 1 College of Horticulture, Northwest Agricultural & Forest University, Yangling, 712100 China; 2 Key Laboratory of Protected Horticultural Engineering in Northwest, Ministry of Agriculture, Shaanxi, Yangling, 712100, China; Hainan University, CHINA

## Abstract

Gamma-aminobutyric acid (GABA) is important in plant responses to environmental stresses. We wished to clarify the role of GABA in maintenance of photosynthesis in muskmelon seedlings (*Cucumis melo* L., cv. Yipintianxia) during saline-alkaline stress. To this end, we assessed the effect of GABA on the structure and function of the photosynthetic apparatus in muskmelon seedlings grown under saline-alkaline stress. These stresses in combination reduced net photosynthetic rate, gas-exchange, and inhibited photosystem II (PSII) electron transport as measured by the JIP-test. They also reduced the activity of chloroplast ATPases and disrupted the internal lamellar system of the thylakoids. Exogenous GABA alleviated the stress-induced reduction of net photosynthesis, the activity of chloroplast ATPases, and overcame some of the damaging effects of stress on the chloroplast structure. Based on interpretation of the JIP-test, we conclude that exogenous GABA alleviated stress-related damage on the acceptor side of PSII. It also restored energy distribution, the reaction center status, and enhanced the ability of PSII to repair reaction centers in stressed seedlings. GABA may play a crucial role in protecting the chloroplast structure and function of PSII against the deleterious effects of salinity-alkalinity stress.

## Introduction

Both saline and alkaline soil conditions are deleterious to many plant species. The effects of salinity, an abiotic stress factor of global importance, are well known. Alkaline conditions have also been shown to inhibit plant growth, to inhibit photosynthesis, to alter metabolism of reactive oxygen species, and to cause cell death [1,2]. Numerous studies have shown that, when both salt and alkaline stresses are present, these deleterious effects are compounded and are more severe than those that result from salinity alone [3,4,5,6]. In tomato seedlings subjected to this combination of stresses, photochemical quenching parameters were reduced, including maximum photochemistry efficiency of photosystem II (PSII). Salinity-alkalinity stress also disrupted the internal lamellar system of granal and stromal thylakoids of tomato seedlings [7].

Chlorophyll a fluorescence (OJIP) is a very small fraction of the dissipated energy from the photosynthetic apparatus. Kautsky and Hirsh [8] first observed the relationship between the primary reactions of photosynthesis and Chl a fluorescence. Upon illumination of darkened plant material, Chl a fluorescence intensity changes in a characteristic pattern [9]. The immediate changes occur in the fast phase and the changes in this phase (transients) are labeled OJIP, which refers to the intensity levels. OJIP transients reflect the redox state of the reaction centers and electron carriers of PSII. Strasserf and Srivastava developed the Chl a fluorescence transient and JIP-test, which is used to relate fluorescence intensity measurements to PSII function [10,11]. The shape of the O-J-I-P fluorescence kinetics, which explores the redox state of the reaction centers and of the donors and acceptors of PSII, is considered as an informative tool for studying the effects of different environmental stresses on photosynthesis [12,13], rather than using only a single parameter F_V_/F_M_ ratio.

Gamma-aminobutyric acid is a four-carbon non-protein amino acid, derived from the decarboxylation of L-glutamate by glutamate decarboxylase in the cytosol [14]. It can also be the product of the catabolism of polyamines [15]. During abiotic stress, GABA serves to stabilize intracellular pH and as a source of carbon and nitrogen for the tricarboxylic acid cycle [16,17]. It was reported that exogenous GABA improved plant growth, enhanced stress tolerance by scavenging free radicals and modulating enzyme activities [17,18,19]. However, to our knowledge, there are no reports on the effect of exogenous GABA on the structure and function of PSII in the response of plants to saline-alkaline stress.

Muskmelon (*Cucumis melon* L.) has high nutritional value and is a profitable crop. It is an important horticultural fruit in China and has been widely cultivated in the northwest. This region, however, has suffered drought and the evaporation that accompanies it, resulting in increased salinity [20]. Muskmelons are sensitive to saline conditions, and, consequently, the muskmelon industry in China has been restricted in recent years. The problem is compounded as, in addition to the neutral salts NaCl and Na_2_SO_4_, the soil in the region contains Na_2_CO_3_ and NaHCO_3_ [21], resulting in soils that are both saline and alkaline.

We have previously shown in muskmelon, that exogenous of GABA accelerated the metabolism of ROS in the chloroplast, stimulated the ascorbate-glutathione cycle, and maintained the permeability of the cell membrane. We concluded that GABA improved the plant’s resistance to saline-alkaline stress and particularly, the resistance of the chloroplast [22]. The main function of chloroplast is to absorb and convert the light for photosynthesis. However, whether exogenous GABA had the ability to protect the photosynthetic system under salinity-alkalinity stress? There is no report about it. Certainly, the mechanism of GABA-mediated protection to resistant saline-alkaline stress is still unidentified. In this study, we will reveal the role of exogenous GABA protected the photosynthetic system under salinity-alkalinity stress with the Chl a fluorescence transient and JIP-test.

## Materials and Methods

### Plant material

Muskmelon seedlings (*Cucumis melo* L., cv. Yipintianxia No. 208) were grown as the method of Hu et al. [17]. Seedlings represent replicates and each index used at least three seedlings for per replicate and three independent experiments were performed.

### Salt-alkaline and GABA treatment

Treatments were started when the transplanted seedlings had four true leaves. Neutral and alkali salts (NaCl:Na_2_SO_4_:NaHCO_3_:Na_2_CO_3_ = 1:9:9:1 molar ratio) were added to the nutrient media for a final concentration of 50 mM. The pH of the final nutrient solution is 8.6. GABA was applied by spraying leaves with 50 mM GABA in water daily; this concentration was chosen based on previous results [17]. There were four treatments: untreated control plants (Control), plants treated with GABA only (CG), plants treated with the complex salts only (S), and plants treated with complex salts and GABA (SG). Seedlings were treated with same amount of GABA and H_2_O at 9:00am every day and continued to be handled for five days. Five days after the plants had been exposed to 50 mM complex salts, the following measurements were made, using the third fully expanded leaf from the shoot apex: net photosynthetic rate, the chl *a* fluorescence transient, and chemical analyses. Same melon lamina with different plants was used to measure chlorophyll fluorescence and gas exchange and analyses ultrastructure. Four times to measure gas exchange and chlorophyll fluorescence for per plant and total used 3 plants for per treatment. We also assessed the structure of the chloroplasts. Samples used for chemical analyses were stored at -80°C until analysis.

### Measurement of photosynthesis

The net photosynthetic rate (P_n_) and gas-exchange parameters were measured with a portable photosynthesis system LI-6400 (Li-COR Inc., USA). Measurements were made at a CO_2_ concentration of 380 ± 10 μmol·mol^-1^, 25°C, and with 800 μmol of photons m^-2^s^-1^ at the surface of the leaf. Stomatal limitation (L_s_) was calculated as L_s_ = 1-Ci /C_a_, where C_i_ and C_a_ represent the intercellular and ambient CO_2_ concentration, respectively [7].

### Measurement of Chl a fluorescence transient and JIP-test parameters

Chl a fluorescence transients were recorded with a Plant Efficiency Analyzer fluorometer (PEA; Hansatech, Ltd., UK) at room temperature. Measurements were carried out at 9:30 am, and leaves for dark adaptation at least 30 min. After that, the leaves exposed to red light of 650 nm from three high intensity light-emitting diodes, which is readily absorbed by the chloroplasts. Light intensity reaching the leaf was 3000 μmol photons·m^-2^·s^-1^, which was sufficient to generate maximal fluorescence for all treatments. Use the program PEA plus 1.0.01 to collecting the data and the Biolyzer 3.0 to calculation of the OJIP test parameters. Data were transferred into Excel (Microsoft, Redmond, USA) for further expound, including the parameters introduced by Strasser et al. [10]. A summary of OJIP test parameters used in this study is shown in Table 1. The fraction of the oxygen evolving complex was compared to the control by calculating according to Appenroth et al. [23] as [1-V_k_/V_J_]_treatment_ / [1-V_k_/V_J_]_control_.

**Table 1:** JIP-test parameters.

**Formulae and terms**	**Illustrations**
Fluorescence parameters	
F_o_ = F_50μs_	Fluorescence intensity at 50 μs
F_300μs_	Fluorescence intensity at 300 μs
F_J_	Fluorescence intensity at 2 ms
F_I_	Fluorescence intensity at 30 ms
F_M_	Maximal fluorescence intensity
W_k_	The ratio of K phase of J phase. W_k_ = F_k_/F_J_
V_J_	Relative variable fluorescence at 2 ms. V_J_ = (F_J_-F_o_)/(F_M_-F_o_)
M_o_	Approximated initial slope of the fluorescence transient. M_o_ = 4(F_300μs_–F_o_) /(F_M_-F_o_)
Ψ_o_	Probability that a trapped exciton moves an electron into the electron transport chain beyond Q_A_^-^. ψ_o_ = ET_o_/TR = 1-V_J_
Quantum efficiency/flux ratios	
φE_o_	Quantum yield for electron transport. ET_o_ /ABS = (1-F_o_ /F_M_)·ψ_o_
φD_o_	Expresses the probability that the energy of an absorbed photon is dissipated as heat. φD_o_ = 1-φP_o_ = F_o_ /F_M_
φP_o_	Maximum quantum yield for primary photochemistry. TR_o_ /ABS = 1-F_o_ /F_M_
Phenomenological energy fluxes [per excited cross-section (CS)]	
ABS/CS_m_	Absorption flux per CS. ABS/CS_m_≈ F_M_
TR_o_/CS_m_	Trapped energy flux per CS. TR_o_ /CS_m_ = φP_o_·(ABS/CS_m_)
ET_o_/CS_m_	Electron transport flux per CS. ET_o_/CS_m_ = φE_o_·(ABS/CS_m_)
DI_o_/CS_m_	Dissipated energy flux per CS. DI_o_/CS_m_ = (ABS/CS_m_)-(TR_o_/CS_m_)
RC/CS_m_	Relative number of active PS II reaction centers per excited cross-section. RC/CSm = φP_o_·(V_J_/M_o_)·(ABS/CS_m_)
Specific energy fluxes [per Q _A_^-^reducing PSII reaction center (RC)]	
ABS/RC	Absorption flux per RC. ABS/RC = M_o_·(1/V_J_) ·(1/φP_o_)
TR_o_/ RC	Trapped energy flux per RC. TR_o_/RC = M_o_·(1/V_J_)
ET_o_/ RC	Electron transport flux per RC. ET_o_/RC = M_o_·(1/V_J_)·ψ_o_
DI_o_/ RC	Dissipated energy flux per RC. DI_o_/ RC = ABS/RC-TR_o_/RC
Performance Index	
PI_ABS_	Performance Index on absorption basis. RC/ABS·[φP_o_/(1-φP_o_)]·[ψ_o_/(1- ψ_o_)]
DF_ABS_	Proton motive force on absorption basis. DF_ABS_ = log(PI_ABS_)

### Ultrastructure of the chloroplast

Leaf samples were cut into discs with an area of approximately 1 mm^2^ and incubated in 4% glutaraldehyde in 0.1 M phosphate buffer (pH 7.4; this buffer was used for the entire preparation of samples for microscopy) overnight. The samples were washed three times, for 15 min each time, with buffer; immersed in 1% osmic acid in buffer for 2 h; and washed again in buffer following the same protocol. The samples were dehydrated in a graded ethanol series (50%, 70%, 90%, and 100%), and then in absolute acetone for 15 min. After dehydration, the samples were embedded in Durcupan ACM epoxy resin (Sigma Aldrich, USA). Ultra-thin sections were cut from the samples and stained with uranium acetate followed by lead citrate. The sections were mounted on copper grids and examined with a JEM-1230 transmission electron microscope (JEOL, Peabody, MA, USA) at an accelerating voltage of 80 kV.

### Isolation of intact chloroplasts

Intact chloroplasts were isolated as described by Shu et al. [24] with slight modification. Ten grams of leaves were homogenized for 5 s in 30 mL of 330 mM sorbitol, 30 mM Mes, 2 mM ascorbate, 0.1% bovine serum albumin (BSA) in 50 mM Tris-HCl adjusted to pH 7.6. The homogenate was filtered through four layers of cheesecloth and centrifuged at 800×*g* for 3 min. The resultant supernatant was centrifuged at 3000×*g* for 5 min and the pellet was resuspended in 3 mL of 330 mM sorbitol, 30 mM Hepes, and 0.2% BSA in Tris, adjusted to pH 7.6. This suspension was mixed with 40–80% (v/v) Percoll density centrifugation medium and centrifuged for 3 min at 3000×*g*. The region between 40 and 80% Percoll contained the intact chloroplasts. All procedures were carried out at 4°C.

### Assay of ATPase activity and measurement of total protein

The activities of H^+^-ATPases (EC 3.6.1.35) in the isolated chloroplasts were determined by measuring the release of inorganic phosphate, as described by Liu et al. [25] with some modification. The reaction mixture (500 μL) for assessing H^+^-ATPase activity contained 150 μL 30 mM Hepes-Tris (pH 6.0), 50 μL of a solution containing 50 mM KCl and 3 mM MgSO_4_, 50 μL 0.1 mM (NH_4_)_2_MoO_4_, 50 μL 50 mM NaNO_3_, 50 μL 0.1 mM Na_3_VO_4_, 50 μL 3 mM ATP, and 100 μL chloroplast suspension. The reaction mixtures were in a water bath at 37°C, after which 50 μL 3 mM ATP-Tris (pH 8.0) was added to start the reaction. The reaction was terminated after 15 min by the addition of 50 μL 55% trichloroacetic acid. After incubate reaction mixture at room temperature for 15 min, then 2.5 mL inorganic phosphorus protectant (containing 0.016 mM EDTA-Na_2_, 4% (NH_4_)_2_MoO_4_, 0.01 mM PVP, 0.172 mM hycroxylatimne sulphate, 0.0875 mM H_2_SO_4_). After 2 min, added 250 μL 6.47 mM NaOH and shaked. The inorganic phosphate concentration of the supernatant was assessed spectrophotometrically at 550 nm after 40 min. To measure Ca^2+^-ATPase (EC 3.6.3.8) activity, the same solution was used except 3 mM Ca(NO_3_)_2_ was added in place of MgSO_4_. Mg^2+^-ATPase (EC 3.6.1.3) activity was measured as described by Cai et al. [26] and Robinson [27], with slight modification. The reaction mixture (0.8 m L) contained 62.5 mM Tris-HCl (pH 8.0), 25 mM NaCl, 6.25 mM MgCl_2_, 55 μM phenazine methosulfate, 12.5 mM dithiothreitol, and 100 μL chloroplast suspensions. The reaction mixtures were illuminated for 10 min in a water bath at 20°C, after which 100 μL 50 mM ATP was added to start the reaction. The reaction was terminated after 5 min by the addition of 200 μL 20% trichloroacetic acid. The reaction mixtures were then centrifuged at 12000 × *g* for 5 min. 2.5 mL inorganic phosphorus protectant (containing 0.016 mM EDTA-Na_2_, 4% (NH_4_)_2_MoO_4_, 0.01 mM PVP, 0.172 mM hycroxylatimne sulphate, 0.0875 mM H_2_SO_4_) added into 0.5 mL supernate. After 2 min, added 250 μL 6.47 mM NaOH and shaked and put for 40 min to assess the inorganic phosphate concentration of the supernatant at 550 nm.

The total protein in the chloroplasts was determined according to the method of Bradford [28], using BSA as standard.

### Statistical analysis

All data presented are the mean values. All experiments were conducted in triplicate and data were analyzed with statistical software SPSS (version 20.0, SPSS Institute, Chicago, USA) using Duncan’s multiple range test at *P*< 0.05 level of significance.

## Results

### Photosynthesis and photosynthetic efficiency of PSII

**Fig 1:**
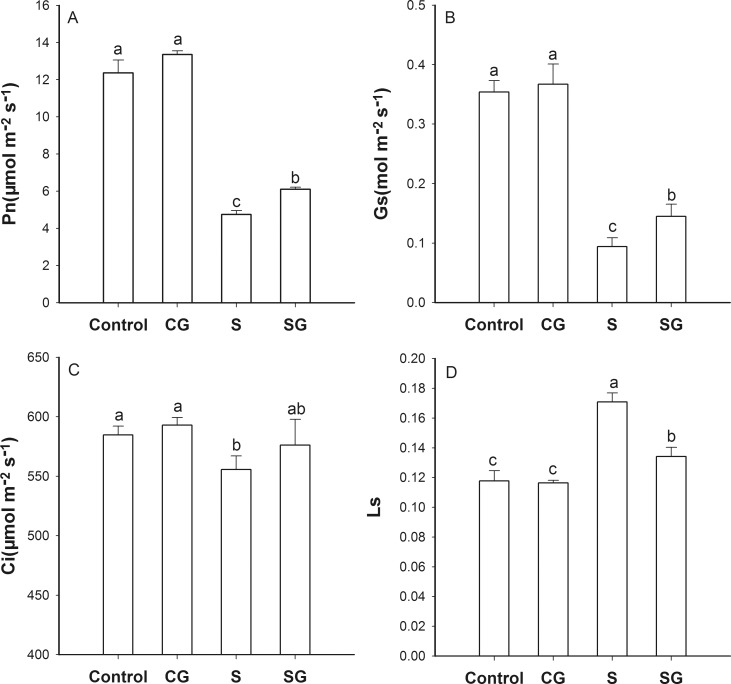
Gas-exchange parameters in muskmelon seedlings. A. Net photosynthetic rate (Pn). B. Stomatal conductance (Gs). C. Intracellular CO2 (Ci). D. Stomatal limitation (Ls). Control, plants grown in medium only; CG, medium with leaf spraying with GABA; S, nutrient medium with complex neutral and alkali salt; SG, medium with both complex neutral and alkali salt and leaf spraying with GABA. Data represent the mean ± SE of three independent experiments (n = 3). Different letters indicate significant differences between treatments (*p* < 0.05).

The net photosynthetic (P_n_), stomatal conductance (G_s_), intercellular CO_2_ concentration (C_i_), and stomatal limitation (L_s_) values in leaves of control plants and plants treated with GABA only were not significantly different (*p* < 0.05) after 5 days of 50 mM complex salts stress treatment. However, in plants exposed to saline-alkaline stress P_n_ declined by 61.6%, G_s_ declined by 73.4%, and L_s_ increased 44.9% compared to the control plants (Fig 1). Foliar application of GABA to stressed plants significantly increased P_n_, G_s_ and C_i_, and significantly reduced (*p* < 0.05) L_s_ as compared to the salinity-alkalinity treatment, partly restoring the effects of stress on these parameters.

### Fast chlorophyll a fluorescence transient

**Fig 2:**
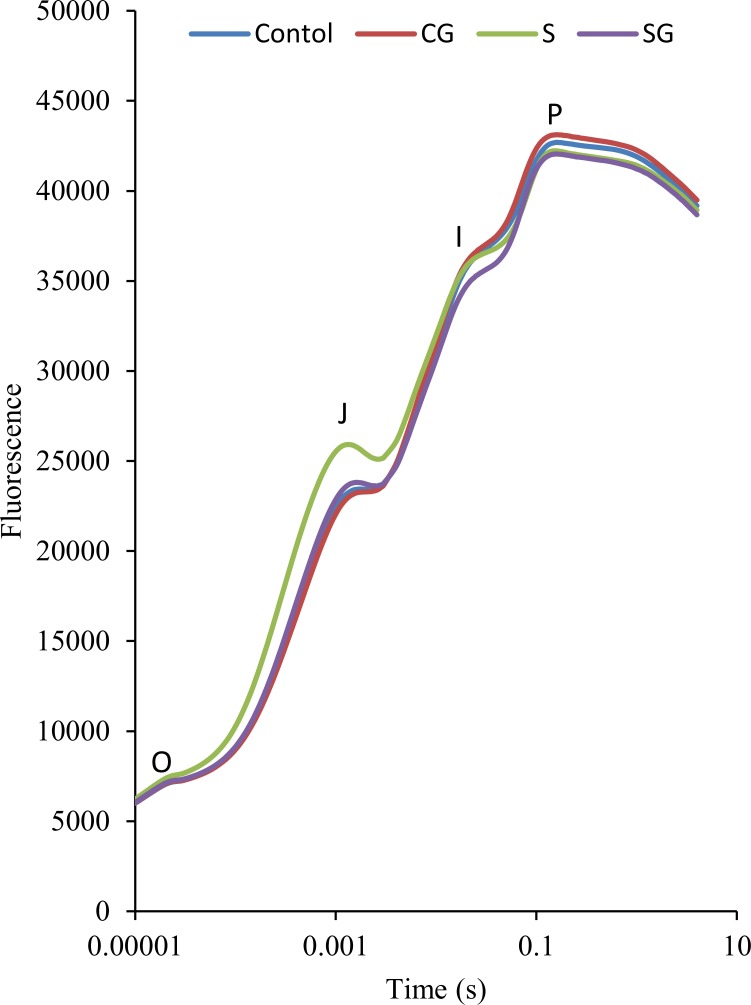
Chlorophyll a fluorescence transients in muskmelon seedlings. Control, plants grown in medium only; CG, medium with leaf spraying with GABA; S, nutrient medium with complex neutral and alkali salt; SG, medium with both complex neutral and alkali salt and leaf spraying with GABA.

The Chl a transient curves for all treatments are presented in Fig 2. Muskmelon seedling leaves would be trapped a saturating light pulse after 30 min dark adaptation, the chlorophyll a fluorescence is form OJIP curve rapidly. After maximum fluorescence, it began to decline. We observed no significant difference in the transient curves of the control and GABA-treated plants. The curve of only complex salts treated plants had 8.4% more fluorescence at the J phase than the control and less fluorescence at the I and P phase. The J phase of stressed plants treated with GABA was no different than the J phase of the control, but the I phase had even less fluorescence (it was further reduced by 2.6%) than the stressed plant (Fig 2).

### The donor side of PSII

**Fig 3:**
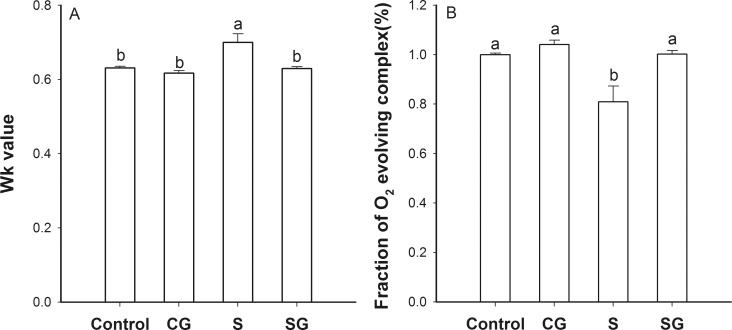
JIP-test parameters on the donor side of PSII in muskmelon seedlings. A. Ratio of the K phase to the J phase (Wk value). B. The fraction of oxygen evolving complex. Control, plants grown in medium only; CG, medium with leaf spraying with GABA; S, nutrient medium with complex neutral and alkali salt; SG, medium with both complex neutral and alkali salt and leaf spraying with GABA. Data represent the mean ± SE of three independent experiments (n = 3). Different letters indicate significant differences between treatments (*p* < 0.05).

An analysis of the fluorescence emission data from the JIP-test yielded values for the ratio of fluorescence intensity of the K phase to that of the J phase (W_k_ values) and the fractions of oxygen evolving complex for all treatments (Fig 3). These values were statistically similar for the control plants and plants treated with GABA alone (*p* < 0.05). Plants exposed to saline-alkaline stress had W_k_ values that were 11.1% greater than the control plants and fraction of oxygen evolving complex values that were 19.0% less than the control; both values were significantly different than the control values (*p* < 0.05). However, stressed plants that had been treated with foliar application of GABA had W_k_ values and fraction of O_2_ evolving complex values that were not different from the control. We suggest that saline-alkaline stress had a negative effect on the donor of the photosynthetic electron transport chain and that GABA alleviated this effect.

### The acceptor side of PSII

**Fig 4:**
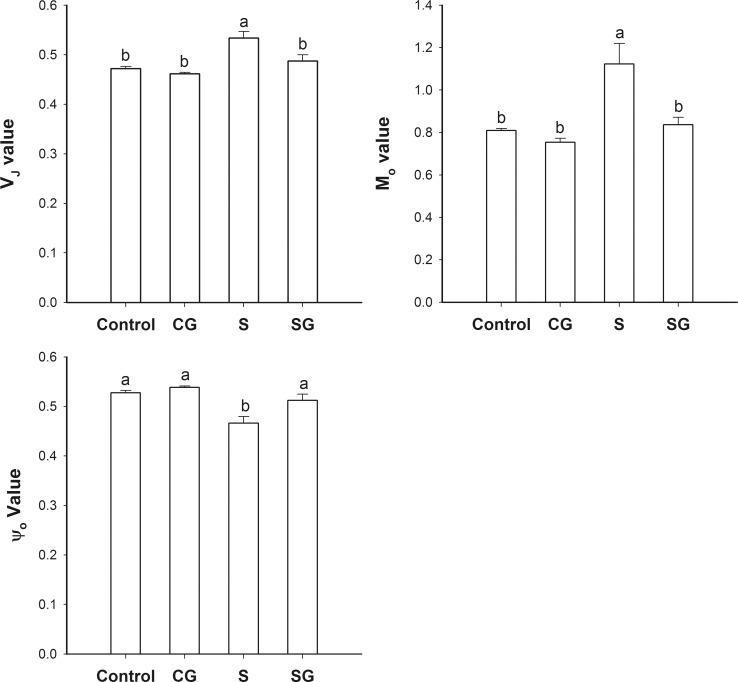
JIP-test parameters on the acceptor side of PSII in muskmelon seedlings. A. Relative variable fluorescence at 2 ms (V_J_). B. Approximated initial slope of the fluorescence transient (M_o_ value). C. Probability that a trapped exciton moves an electron into the electron transport chain beyond QA (ψ_o_). Control, plants grown in medium only; CG, medium with leaf spraying with GABA; S, nutrient medium with complex neutral and alkali salt; SG, medium with both complex neutral and alkali salt and leaf spraying with GABA. Data represent the mean ± SE of three independent experiments (n = 3). Different letters indicate significant differences between treatments (*p* < 0.05).

Fig 4 shows the values for relative variable fluorescence at 2 ms (V_J_), the approximated initial slope of the fluorescence transient (M_o_ value), and the probability that a trapped exciton moves an electron into the electron transport chain beyond plastoquinone A (Q_A_; the probability is ψ_o_). These fluorescence parameters provide information about the acceptor side of PSII. V_J_ and M_o_ values were somewhat lower, and ψ_o_ value was slightly greater, in plants treated only with GABA than in the control plants, although the difference was not significant (*p* < 0.05). V_J_ and M_o_ values of saline-alkaline stressed plants were 13.1% and 38.8% greater, while the ψ_o_ value was 11.5% less, in the stressed plants than in the control (significant differences at *p* < 0.05). These results are consistent with the interpretation that saline-alkaline stress inhibited photosynthetic electron transport. Stressed plants that were treated with GABA had lower value of V_J_, M_o_, and higher value of ψ_o_ that were significantly different (*p* < 0.05) than the only stress values.

### The energy distribution

**Fig 5:**
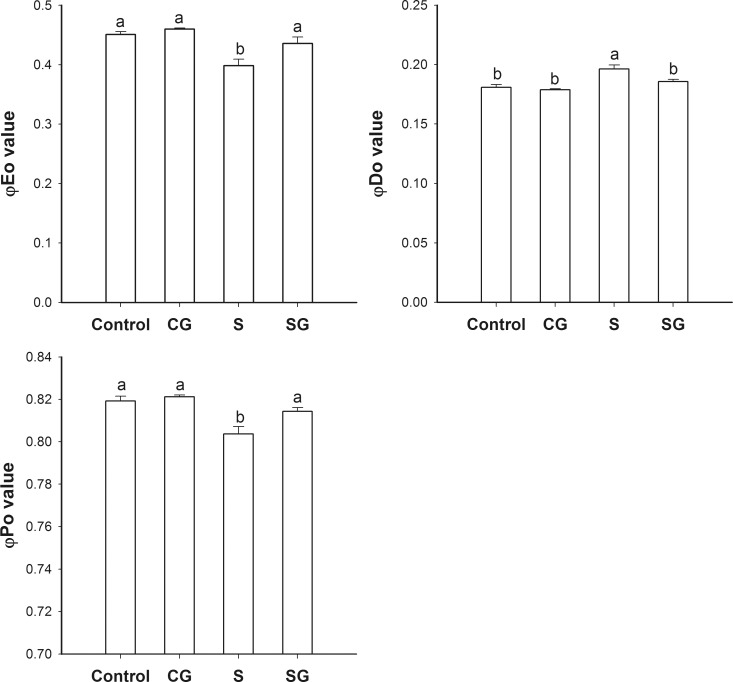
Energy distributions during photosynthesis in muskmelon seedlings. A. Quantum yield for electron transport (φE_o_). B. The probability that the energy of an absorbed photon is dissipated as heat (φD_o_). C. Maximum quantum yield for primary photochemistry (φP_o_). Control, plants grown in medium only; CG, medium with leaf spraying with GABA; S, nutrient medium with complex neutral and alkali salt; SG, medium with both complex neutral and alkali salt and leaf spraying with GABA. Data represent the mean ± SE of three independent experiments (n = 3). Different letters indicate significant differences between treatments (*p* < 0.05).

The measurements for quantum yield for electron transport (φE_o_), an expression for the probability that the energy of an absorbed photon is dissipated as heat (φD_o_), and the maximum quantum yield for primary photochemistry (φP_o_) are presented in Fig 5. Under normal conditions, φE_o_, φD_o_, and φP_o_ values in leaves of untreated and GABA-treated plants were relatively similar. The φE_o_ and φP_o_ values in plants subjected to saline-alkaline stress were 11.7% and 2.0% less than the control, respectively, while the φD_o_ value was increased 8.8% relative to the control. Under stress condition, the addition of exogenous GABA alleviated the increase of φD_o_ value and the reduction of φE_o_ and φD_o_ to control levels.

### The reaction center status

The parameters relate to the status of the reaction centers of the chloroplast of the experimental plants are reported in S1 Table. GABA treatment by itself did not affect these parameters as compared to the control. Saline-alkaline stress reduced DI_o_/CS_m_, ET_o_/CS_m_, and RC/CS_m_ values, and increased ABS/RC, DI_o_/RC, TR_o_/RC, and ET_o_/RC values. GABA treatment of the stressed plants resulted in partial, although not significant, reversal of the stress-induced increase of ABS/RC, TR_o_/RC, and ET_o_/RC values and significantly increased the RC/CS_m_ value (*p* < 0.05).

### The performance index and driving force

**Fig 6:**
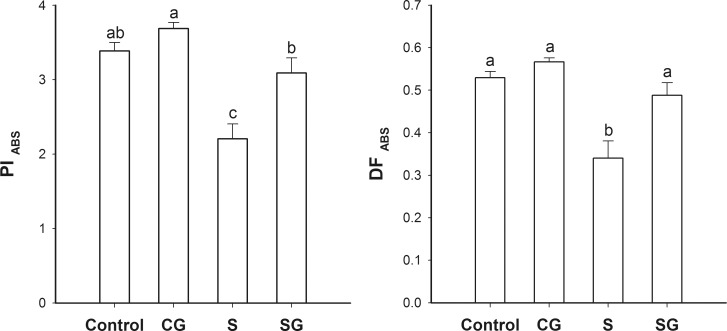
Two performance indices in muskmelon seedlings. A. Performance index on an absorption basis (PI_ABS_). B. Proton motor force on an absorption basis (DF_ABS_). Control, plants grown in medium only; CG, medium with leaf spraying with GABA; S, nutrient medium with complex neutral and alkali salt; SG, medium with both complex neutral and alkali salt and leaf spraying with GABA. Data represent the mean ± SE of three independent experiments (*n* = 3). Different letters indicate significant differences between treatments (*p* < 0.05).

The performance index and driving force are presented, expressed on an absorption basis (PI_ABS_ and DF_ABS_, respectively), in Fig 6. There was no significant difference in these values in plants treated with GABA alone and the untreated control plants (*p* < 0.05). Plants under saline-alkaline stress had PI_ABS_ and DF_ABS_ that were 34.8% and 35.7% less than the control, respectively. When the stressed plants were treated with GABA, PI_ABS_ and DF_ABS_ values increased by 39.8% and 43.5%, respectively, compared to the stressed plants not treated with GABA. In the case of DF_ABS_, there was no significant difference between the stressed, GABA-treated plants and the control (*p* < 0.05).

### Activity of ATPase

**Fig 7:**
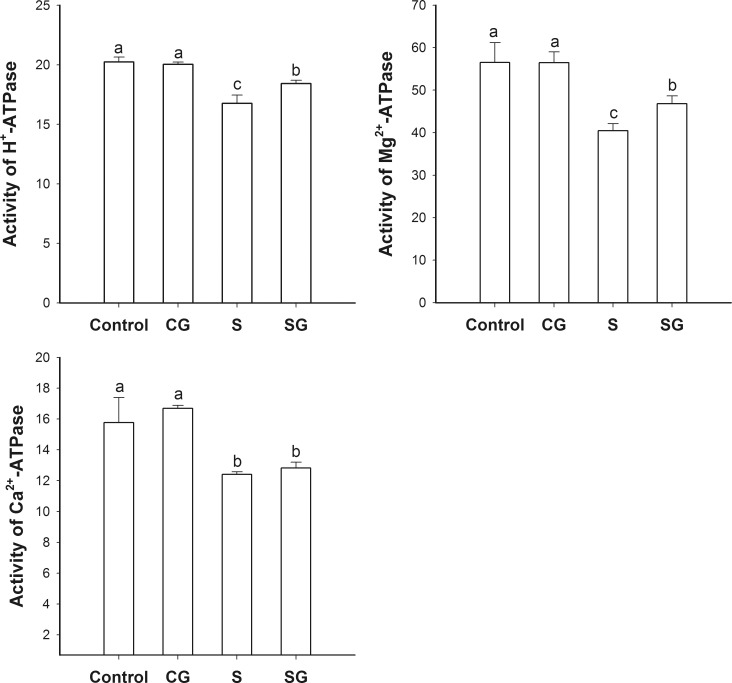
Activity of ATPases in chloroplasts isolated from muskmelon seedlings. Activity of: A. H^+^-ATPase, B. Mg^2+^-ATPase, C. Ca^2+^-ATPase. Control, plants grown in medium only; CG, medium with leaf spraying with GABA; S, nutrient medium with complex neutral and alkali salt; SG, medium with both complex neutral and alkali salt and leaf spraying with GABA. Data represent the mean ± SE of three independent experiments (n = 3). Different letters indicate significant differences between treatments (*p* < 0.05).

There was no significant difference in the activity of the different ATPases that were assessed in the chloroplasts of leaves from control or GABA-treated plants (*p* < 0.05), as shown in Fig 7. In leaves from saline-alkaline stressed plants, the activities of H^+^-ATPase, Mg^2+^-ATPase, and Ca^2+^ATPase were decreased by 17.2%, 21.3% and 28.4%, respectively, compared to the control. Fig 7 also shows that these activities were greater in stressed plants treated with GABA than in the only stressed plants, although the activities were still lower than in the control plants. We conclude that exogenous GABA maintained the activities of these three ATPase, in plants exposed to saline-alkaline stress.

### Ultrastructure of the chloroplast

**Fig 8:**
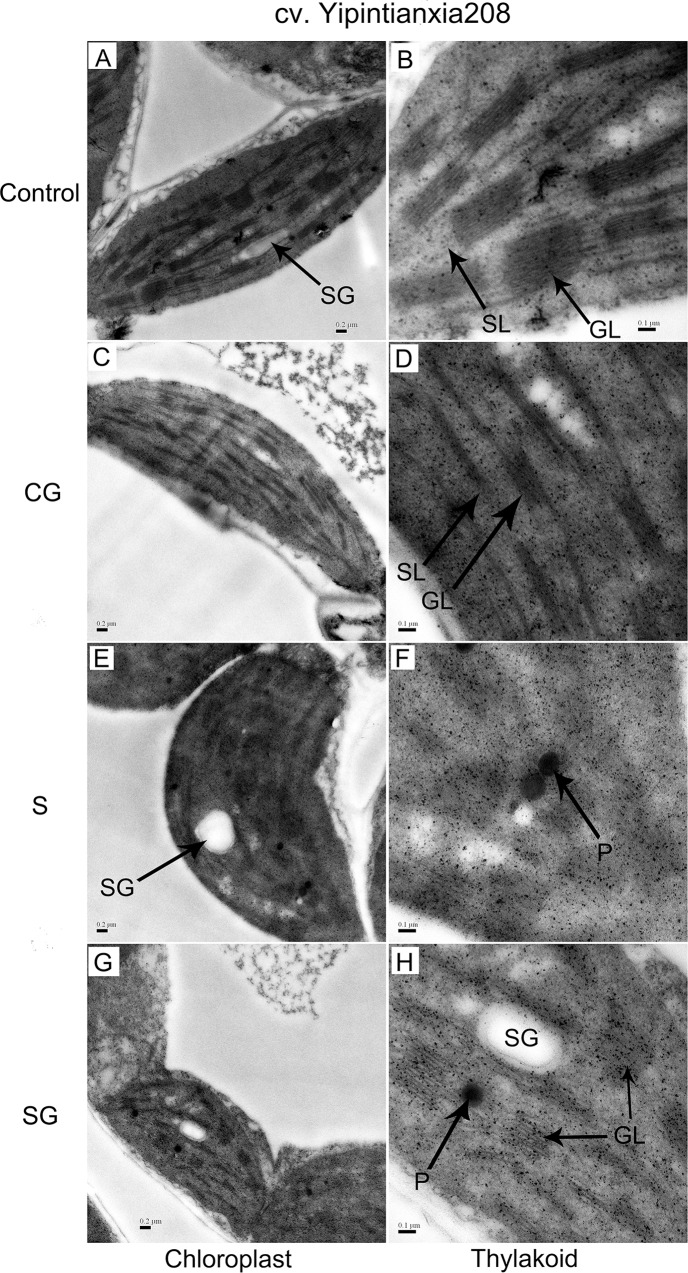
Photomicrographs of chloroplasts isolated from muskmelon seedlings. Control, plants grown in medium only; CG, medium with leaf spraying with GABA; S, nutrient medium with complex neutral and alkali salt; SG, medium with both complex neutral and alkali salt and leaf spraying with GABA. SL, stroma lamellae; GL, grana lamellae; SG, starch grains; P, plastoglobuli. Scale bars for chloroplasts and thylakoids are 0.2μm and 0.1μm, respectively.

Fig 8 shows the microscopic structure of chloroplasts from control and treated plants. Chloroplasts from control and GABA-treated plants were intact with structural integrity and had a typical elongated shape and a regular arrangement of granal and stromal thylakoids (Fig 8A–8D). The chloroplasts from plants treated with saline-alkaline stress were markedly different in appearance: they were swollen compared to the control and the membranes of the granal and stromal thylakoids were indistinct under the microscope (Fig 8E and 8F). Chloroplasts from stressed plants that received exogenous GABA had more normal appearing chloroplasts that those from stressed plants (Fig 8G and 8H). Specifically, the images reveal that the internal lamellae of these thylakoids were better integrated than those from stressed plants not treated with GABA. Apparently, exogenous GABA maintained the stability of photosynthetic apparatus during saline-alkaline stress.

## Discussion

Saline stress often decreases the photochemical efficiency of plants, which has been ascribed to suppression of PSII activity [29]. The relationship between L_s_ and non-stomatal factors has been evaluated by [30]. When both C_i_ and G_s_ decrease, P_n_ is limited by stomatal conductance. When G_s_ decreases and C_i_ either does not change or increases, the reduced P_n_ that is observed may be due to non-stomatal factors. In this study, saline-alkaline stress reduced P_n_ and G_s_, and increased L_s_ (Fig 1). This suggests that stress-induced limitation of photosynthesis in this study was mainly unrelated to effects on the stomata. Exogenous GABA supplied to saline-alkaline stressed plants alleviated stress-induced reductions in P_n_, G_s_ and Ci, and increased L_s_. This suggests that exogenous GABA absorbed by plants could act as a temporary nitrogen source and osmotic substances which reduced reduce the injury of mesophyll cells under saline-alkaline stress. Meanwhile, GABA may be involved in regulation of ABA levels in plant and maintain higher P_n_ levels by enhancing the level of stomatal openness. We interpret these results as evidence that saline-alkaline stress blocked electron transport, which in turn damaged the photosynthetic apparatus (Fig 8), and that exogenous GABA alleviated these effects.

Photosynthesis, the process of changing light energy to chemical energy, is critical in the growth and development of plants. In this study, saline-alkaline stress altered the chl a fluorescence transient (Fig 2) and reduced the maximum quantum yield for primary photochemistry (Fig 5). This is evidence that photoinhibition occurred, on both the acceptor and donor side of PSII [31]. The W_k_ value assesses the activity of the oxygen evolution complexes on the donor side of PSII and an increase in W_k_ indicates damage to the complexes [9]. In this study, we can observe that stress increased the W_k_ value (Fig 3A), which indicative of damage to the donor side of PSII. Specifically, the decrease of fraction of O_2_ evolving complex further more supported it (Fig 3B). V_J_ represents the closed degree of the reaction center at 2ms, M_o_ represents the maximum reduction rate of Q_A_, and ψ_o_ represents the probability that a trapped exaction moves an electron into the electron transport chain beyond Q_A_^-^ [10]. The values of V_J_ and M_o_ increased and the value of ψ_o_ decreased under stress, which consistent with blockage of electron transfer from the primary acceptor Q_A_ to the secondary acceptor plastoquinone B (Q_B_) on the acceptor side of PSII (Fig 4). φE_o_, the probability that an absorbed photon leads to an electron transport farther than Q_A_, and φP_o_, the probability that an absorbed photon leads to a reduction of Q_A_^-^ and φD_o_, the probability that the energy of an absorbed photon was dissipated. Stress induced the reduction of φE_o_ and φP_o_ and increase of φD_o_, these variation illuminated stress can re-distribution of the quantum efficiency of PSII (Fig 5). As S1 Table shows, stress had no difference on absorbing and trapping energy fluxes per excited cross section (ABS/CS_m_, TR_o_/CS_m_), but significantly affected the electron transport flux and dissipated energy flux (ET_o_/CS_m_, DI_o_/CS_m_). Stress also significantly increased the specific energy fluxes per reaction center (ABS/RC, DI_o_/RC, TR_o_/RC, and ET_o_/RC). These may be due to the reduction of the relative number of active PSII reaction centers per excited cross-section (RC/CS_m_). Stressed plants treated with GABA alleviated the increase of W_k_, V_J_, M_o_, φD_o_, ABS/RC, DIo/RC, TRo/RC, and ETo/RC, or decrease Fraction of O_2_ evolvingcomplex, ψ_o_, φE_o_, φP_o_of ET_o_/CS_m_, DI_o_/CS_m_ and RC/CS_m_. These indicated that exogenous GABA reduced the heat dissipation and distributed more energy for transfer electron, and promoted probability that an absorbed photon lead to an electron transport further than Q_A_. Hu et al. [17] research showed exogenous GABA with stressed plants could further more increase the endogenous GABA. The GABA accumulation has two pathways. One is converted glutamate to succinate via GABA that called the GABA shunt [19]. Another is come from polyamines (PAs) oxidation [16, 32]. In our study, exogenous GABA alleviation effect of stressed plants may though the GABA shunt to promoting the tricarboxylic acid cycle (TCA) and ensuring the operation of photosynthetic electron transport chain. In addition, exogenous GABA may mediate the PAs metabolism to improving tolerance of salinity-alkalinity stress. The polyamines could enhance antioxidant, increase ATP synthase, Qb, psbA protein and the saturated fatty acid contents of thylakoid membranes, decrease the content of D2 protein and LHCII type III in NaCl-stressed thylakoid membranes to overcoming the damaging effects of stress on the structure and function of the photosynthetic apparatus and improving the photochemical efficiency of PSII of the salt stressed plants [24, 33, 34].

The generation of ATP is the last step in the light reactions of photosynthesis. In this study, saline-alkaline stress inhibited the activity of three ATPases: the H^+^-ATPase, the Mg^2+^-ATPase, and the Ca^2+^-ATPase (Fig 7) and this inhibition was partly reversed by GABA. It has been reported that GABA could regulate cytosolic pH by consuming a proton and it could also function as a nitrogen source to promote the tricarboxylic acid cycle [17]. This effect of GABA might improve ATPase function [17]. Another possiility is that exogenous GABA could affect the endogenous GABA levels that stimulated CaM-dependent GAD activity [17] and amplifyed the GABA accumulation/Ca^2+^ release cycle. The observed effects of stress and GABA on the driving force (DF_ABS_ value) support this suggestion (Fig 6).

In higher plants, the photosynthetic machinery is mainly localized in the thylakoids membranes of the chloroplasts. Intact thylakoids are essential to efficient photosynthesis and the structure of thylakoids is a major factor that affects functionality and efficiency of the photosynthetic apparatus. Saline stress reduces photosynthetic efficiency and electron transport; it may be that this inhibition is due to effects of stress on the structure of photosynthetic apparatus [35]. In this study, we detected degradation of thylakoid membranes accompanied by the accumulation of plastoglobuli under saline-alkaline stress (Fig 8E and 8F). Plants exposed to environmental stress accumulate large quantities of reactive oxygen species (ROS) in their chloroplasts [2]. In this study, we suggest that inactivation of the electron transport chain prevented the alternate oxidation and reduction of P680, leading to increased oxidation potential on the donor side of PSII (Fig 3). This could, in turn, cause peroxidation of the chloroplast membrane and other structural abnormalities (Fig 8). Additionally, the prevention of electron transfer from the primary acceptor Q_A_ to the secondary acceptor Q_B_ would form the triplet chlorophyll (^3^P680) which, when combined with oxygen, would generate singlet oxygen (Fig 4). The singlet oxygen could cause D1 protein, important in repair, to be degraded and cause pigment decomposition, directly inhibiting photosynthesis [36, 37]. GABA, which appears to stabilize the photosynthetic electron transport chain and the photosynthetic apparatus (Fig 8), may enhance the activity of superoxide dismutase, accelerate ascorbic acid-glutathione cycling, and increase the content of antioxidants that scavenge ROS, leading to stabilization of the structure and function of chloroplast[22, 38]. The similar results with Malekzadeh [39], who indicated that exogenous GABA by enhancing some antioxidant enzymes activity and reducing MDA content to alleviate the damage of tomato seedlings under chilling stress.

## Supporting Information

S1 Table Status of the reaction centers in chloroplasts of muskmelon seedlings. Control, plants grown in medium only; CG, medium with leaf spraying with GABA; S, nutrient medium with complex neutral and alkali salt; SG, medium with both complex neutral and alkali salt and leaf spraying with GABA. Data represent the mean ± SE of three independent experiments (n = 3). Different letters indicate significant differences between treatments (*p* < 0.05).
(DOCX)
