# Supplementary material for: Exogenous γ-Aminobutyric Acid Improves the Structure and Function of Photosystem II in Muskmelon Seedlings Exposed to Salinity-Alkalinity Stress
Source: PLoS One. 2016 Oct 20;11(10):e0164847. doi: 10.1371/journal.pone.0164847 (PMC5072714; doi:10.1371/journal.pone.0164847)
Supplement: S1 Table — Control, plants grown in medium only; CG, medium with leaf spraying with GABA; S, nutrient medium with complex neutral and alkali salt; SG, medium with both complex neutral and alkali salt and leaf spraying with GABA. Data represent the mean ± SE of three independent experiments (n = 3). Different letters indicate significant differences between treatments (p < 0.05). (DOCX) [file pone.0164847.s001.docx]

**S1 Table. Status of the reaction centers in chloroplasts of muskmelon seedlings.**

| Index | Control | CG | S | SG |
| --- | --- | --- | --- | --- |
| ABS/CS_m_ | 42683.3±498.9a | 43111.3±720.5a | 42213.3±398.9a | 42048.6±133.2a |
| TR_o_/CS_m_ | 36492.3±494.4a | 36830.7±663.9a | 36084.6±432.7a | 35771.3±127.3a |
| ET_o_/CS_m_ | 19257.3±350.7ab | 19825.3±253.5a | 16811.7±311.7c | 18327.3±501.8b |
| DI_o_/CS_m_ | 6191.0±22.2ab | 6280.7±56.6a | 6128.6±57.2b | 6277.3±20.95a |
| RC/CS_m_ | 21298.7±412.7a | 22569.7±372.4a | 17296.2±788.9b | 20887.9±415.6a |
| ABS/RC | 1.946±0.018b | 1.856±0.039b | 2.355±0.127a | 1.946±0.032b |
| TR_o_/RC | 1.664±0.012b | 1.585±0.034b | 2.014±0.113a | 1.655±0.026b |
| ET_o_/RC | 0.878±0.010b | 0.853±0.016b | 0.936±0.025a | 0.847±0.013b |
| DI_o_/RC | 0.282±0.006b | 0.270±0.005b | 0.342±0.014a | 0.291±0.006b |
